# 

**Published:** 1904-09

**Authors:** 


					﻿CONTENTS.
ORIGINAL COMMUNICATIONS—
Enterocolitis, Cholera Infantum, Dysentery and Painful Dentition. By E. Vxn Goidtsnoven, $
M.D , Atlanta, Ga........................................................ 3fl
Simple Method of Home Modification of Milk and Its Application in Infant Feeding. By
Louis C. Rouglin, M.D., Atlanta, Ga...................................... 371;
The Relationship of Albuminuria to The Life Insurance Company. By W. E. Wilmerding, M.D.,
Atlant*, Ga....................................................:.......... 37a
EDITORIAL NOTES AND COMMENTS—
The Safety of Chloroform...............................................  38(1
The Statesboro Lynching.................................................... 33$
The Commission for the Study of Tuberculosis............................. 3^3
NEWS AND NOTES...............................................................   g8|!
CONTENTS CONTINUED..............................................................X
CONTENTS— CONTINUED.
BOOK REVIEWS—
A System of Practical Surgery, (von Bergmann, Bruns and von Mikulicz)...... 886
The Surgery of the Heart and Lungs. (Rickets)..................... 386
SELECTIONS AND ABSTRACTS—
Louis Pasteur....................................................  387
The Relations of the Nursing Profession to that of Medicine and to Society. 395
Medical Treatment of Diabetes..................................... 404
Ocular Lesions in Scarlatina...................................... 413
Some Advice to Give, and Some Not to Give, the Tuberculous........ 41
Modern Treatment of Cholera Infantum.............................. 422
Chorea and Anemia ...............................................  425
Appendicitis from the Standpoint of the Physician................. 427
Dr. Pettey’s Western Retreat...................................... 429
Cause and Treatment of Rheumatism...............................   430
Pathology and Treatment of Simple Fracture of the Patella........ 431
Facial Neuralgia.................................................. 431
Annual Session Mississippi Valley Medical Association............  432
MEDICAL ITEMS—Continued....................xviii, xx, xxii, xxiv, xxvi, xxviii, xxxv.
				

